# Cutaneous Squamous Cell Carcinoma as a Metastasis From Invasive Breast Cancer: A Case Report

**DOI:** 10.7759/cureus.77064

**Published:** 2025-01-07

**Authors:** Binod Kumar, Kiran Kumre, Ratan Kumar, Tulika Anand, Kaushik Saha

**Affiliations:** 1 Department of Dermatology, Tata Main Hospital, Jamshedpur, IND; 2 Department of Pediatrics, Manipal Tata Medical College and Tata Main Hospital, Jamshedpur, IND; 3 Department of Dermatology, Manipal Tata Medical College, Jamshedpur, IND; 4 Department of Pathology, Tata Main Hospital, Jamshedpur, IND

**Keywords:** bowen’s disease, chemotherapy, metastasis, non-melanoma, radiotherapy, squamous cell carcinoma

## Abstract

Cutaneous squamous cell carcinoma (cSCC) is a non-melanoma (keratinocyte) skin cancer. cSCC can occur as primary cSCC, second primary cancer (SPC), or metastasis. Histopathology and genetic or molecular studies are used to differentiate SPC from metastasis. Cutaneous metastasis (CM) is often misdiagnosed as a benign skin lesion. However, with early diagnosis and timely treatment, CM from breast cancer carries a good prognosis. Although surgery is the first-line treatment for cSCC, metastatic lesions respond well to chemotherapy and immunotherapy. Here, we present the case of a 73-year-old woman who presented to a dermatologist with a localized skin lesion. After detailed radiological and histopathological investigations, she was diagnosed with cSCC metastasis from breast cancer. With timely diagnosis, her CM responded well to chemotherapy.

## Introduction

Skin cancers are of two types: melanoma and non-melanoma. Non-melanoma includes basal cell carcinoma (approximately 80% incidence), squamous cell carcinoma (SCC) (approximately 20% incidence), and Merkel cell carcinoma (rare but aggressive type) [1]. Cutaneous squamous cell carcinoma (cSCC) can manifest classically as in situ (Bowen’s Disease), keratoacanthoma, or an invasive form [2].

cSCC results from intraepidermal dysplasia, leading to uncontrolled proliferation of atypical epidermal keratinocytes. Risk factors for cSCC include ultraviolet (UV) radiation exposure, advancing age, male sex, fair complexion, immunosuppression, history of previous skin cancer, smoking, and genetic factors. cSCC can be a manifestation of metastasis from a primary tumor elsewhere. It has a good prognosis, with a five-year survival of ≥90% [3].

## Case presentation

A 73-year-old female patient visited the Skin Outpatient Department (OPD) with chief complaints of multiple localized erythematous tender nodules present over the right arm for five months and discharge from the lesions for four days. Other significant history included a lump in the right breast for the last three years, which has now become ulcerative, and aspiration of an abscess from the left lobe of the liver two years ago. The associated comorbidities in this patient were diabetes mellitus (DM), hypertension (HTN), hyperuricemia, dilated cardiomyopathy (DCMP), and cardiac dysfunction. For these comorbidities, she was on carvedilol, furosemide, spironolactone, losartan, and allopurinol.

**Figure 1:**
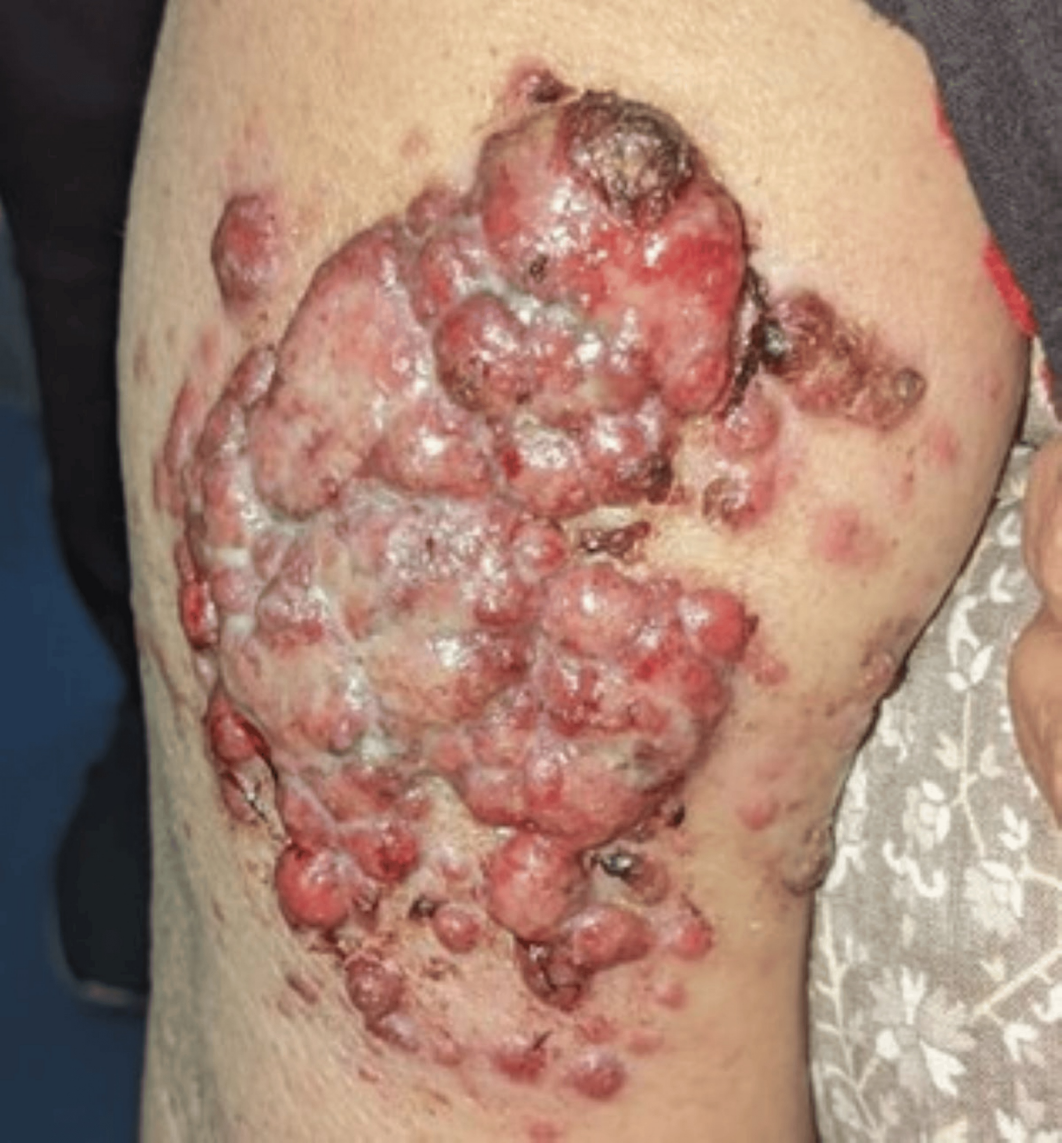
Multiple hemorrhagic nodulo-ulcerative lesions present over extensor aspect of the right arm

On local examination, multiple erythematous and hemorrhagic papulonodular lesions were present over the extensor aspect of the right arm, extending to include an area of 8 x 6 cm (Figure 1). On palpation, tenderness was present and was associated with bleeding. The regional lymph nodes (LNs) were not enlarged.

Local examination of the breast revealed an ulcerative, tender lesion in the right breast with an underlying firm nodular mass 6 x 8 cm in size.

Imaging and pathology reports of right arm lesion

**Figure 2:**
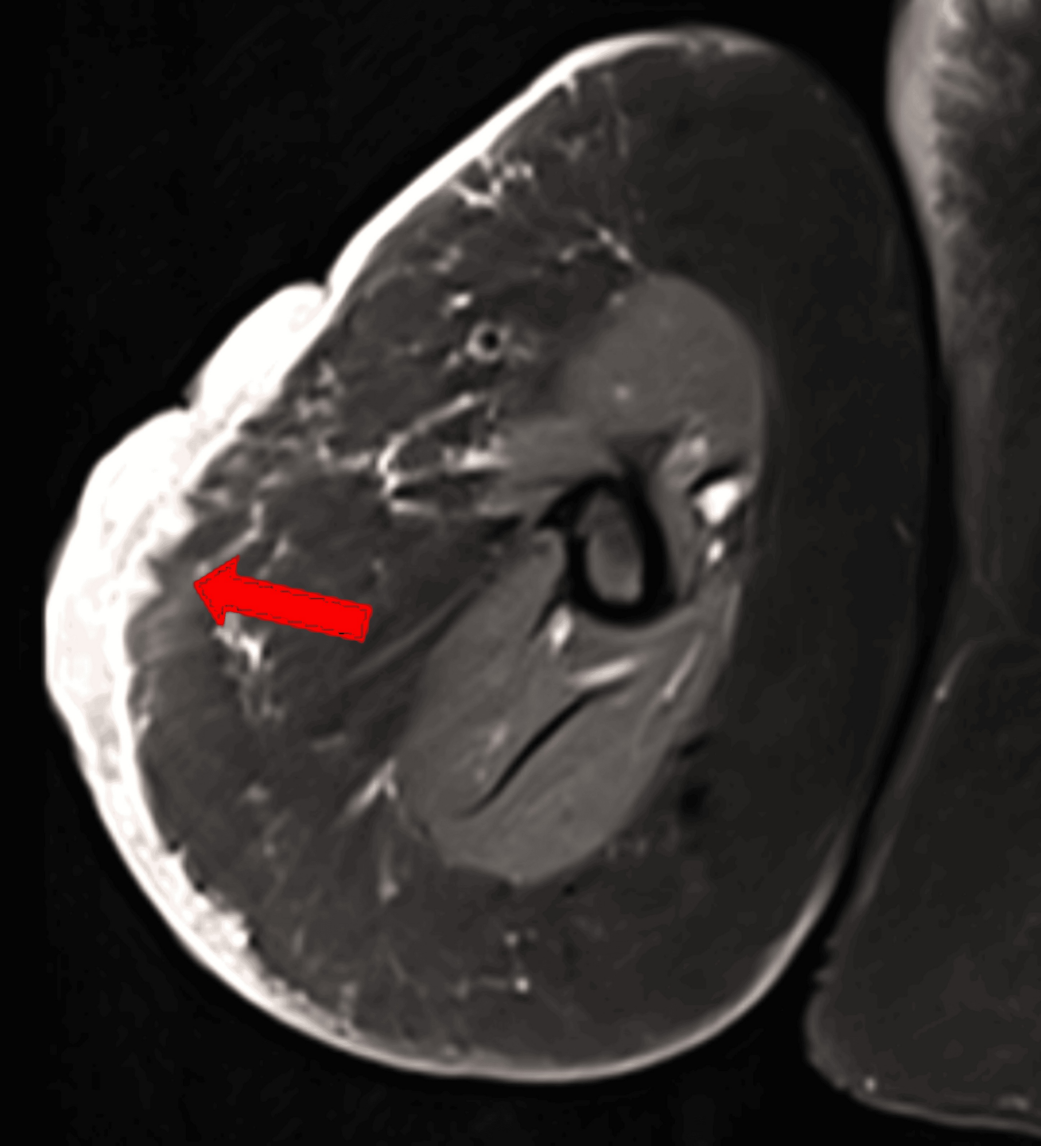
MRI showing an ill-defined lesion in lateral aspect of midarm in cutaneous and subcutaneous plane (arrow) MRI, Magnetic resonance imaging

Magnetic resonance imaging (MRI) of the right arm was done with T1, T2, proton density fat saturation (PDFS), gradient echo (GRE), and short tau inversion recovery (STIR) sequences, which showed an ill-defined lesion in the cutaneous and subcutaneous plane of the midarm on the lateral aspect (Figure 2), with a maximum thickness of 1.2 cm and involving an area of 7 x 8 cm, with no restriction of diffusion, while inhomogeneous enhancement was noted on contrast study. The marrow signal of the humerus and neurovascular bundles were within normal limits. The Doppler study of the vessels in the arm was normal.

**Figure 3:**
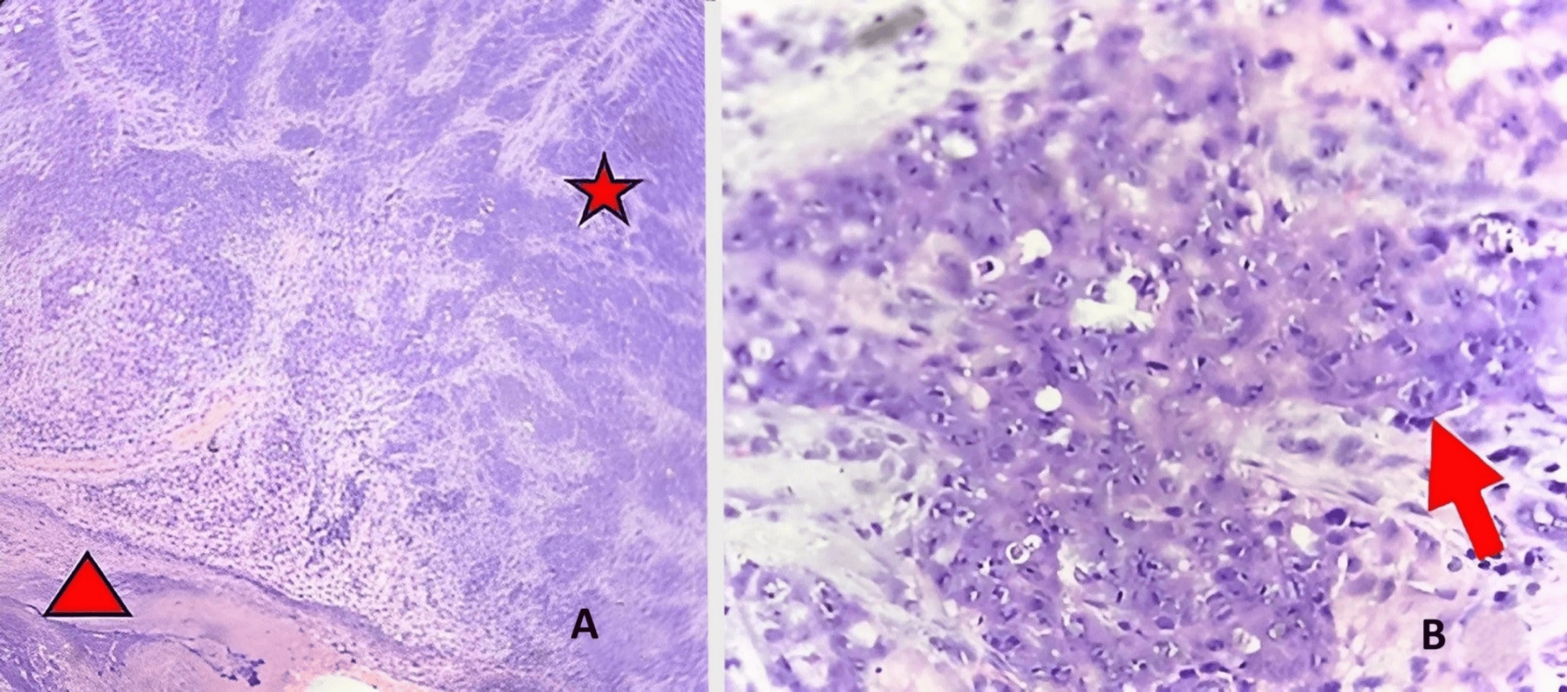
Histopathology slide description (A) Low-power magnification shows skin ulceration (arrowhead) and subepithelial pleomorphic epithelial (ductal) cells, seen in variable sheets (asterisk) and groups, invading the subepithelial stromal tissue. No dysplasia in the overlying epithelium is noted. (B) High-power image shows pleomorphic ductal epithelial cells with marked pleomorphism, prominent nuclei, and abundant eosinophilic cytoplasm (arrow).

Incisional biopsy of the lesion in the right arm (Figure 3) favored a diagnosis of poorly differentiated invasive SCC.

Pathology reports of right breast lesion

**Figure 4:**
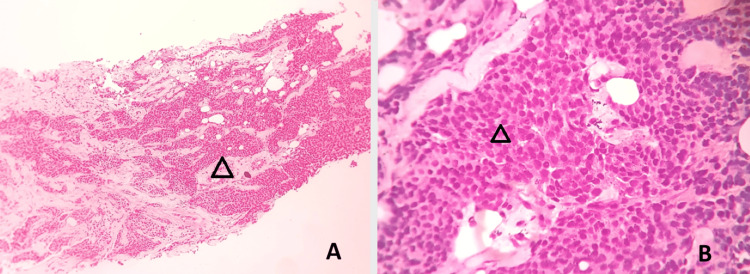
Biopsy of the right breast lump (A) Low magnification (100x) showing infiltrating islands and cords (hollow arrowhead) of neoplastic cells infiltrating into the stroma (hematoxylin and eosin stain, 10x objective, 10x optics). (B) High magnification (400x) showing no tubule formation and moderately pleomorphic cells (hollow arrowhead) with high mitosis, reported as grade 3 according to the Nottingham histological score (hematoxylin and eosin stain, 40x objective, 10x optics).

A core biopsy from the right breast lump (Figure 4) revealed metaplastic carcinoma (a combination of invasive ductal carcinoma (50%) and keratinizing SCC (50%)). The immunohistochemistry (IHC) breast panel showed poorly differentiated invasive ductal carcinoma (Grade III, Ki67 50%, negative Allred score (0/8), human epidermal growth factor receptor 2-negative (HER2-negative) (0/3)).

**Figure 5:**
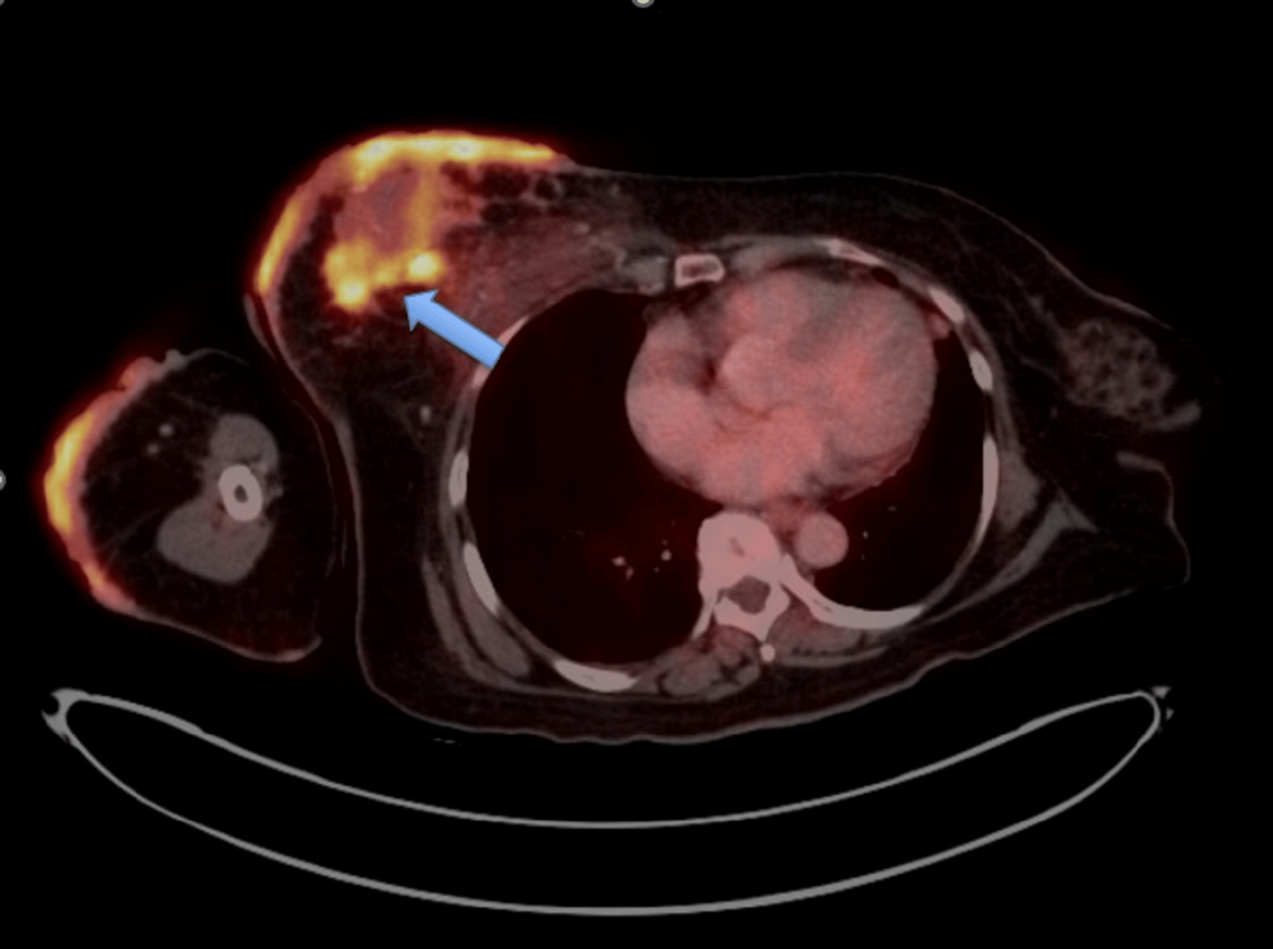
PET scan showing FDG avid contiguous and discrete enhancing soft tissue lesions in the right breast (arrow) PET, Positron emission tomography; FDG, Fluorodeoxyglucose

Positron emission tomography-computed tomography (PET-CT) scan revealed ill-defined fluorodeoxyglucose (FDG)-avid contiguous and discrete enhancing soft tissue lesions in the upper and lower quadrants of the right breast (Figure 5), with a large soft tissue mass lesion in the midline upper quadrant (3.4 x 5.5 cm in axial diameter); FDG-avid nodular enhancing cutaneous soft tissue thickening in the right arm; FDG-avid left supraclavicular LNs, right axillary LNs, right internal mammary LNs, right lower paratracheal, and subcarinal LNs; FDG-avid nodular opacities in the lingular posterior segment of the left upper lobe and the anterior segment of the right upper lobe of the lung; no abnormal FDG uptake noted in the brain, abdomen and pelvis, or musculoskeletal system.

**Figure 6:**
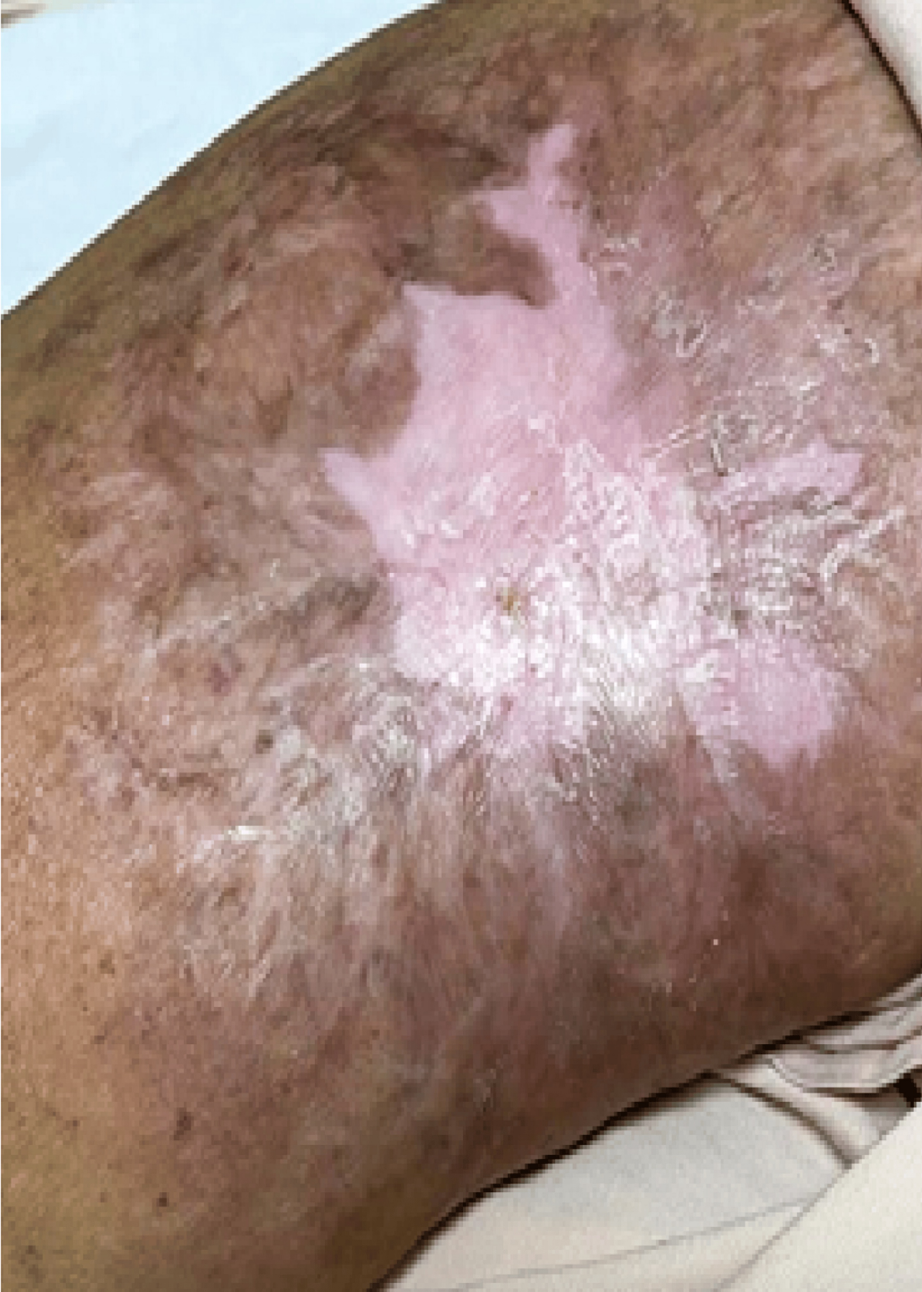
Cutaneous metastatic lesions in arm disappeared after chemotherapy resulting in scarred plaque

After all radiological and pathological investigations, the final diagnosis was “malignant skin adnexal tumor with apocrine differentiation over metastatic carcinoma of the right breast,” triple-negative breast cancer (TNBC), T4B N3M1. She was started on weekly chemotherapy with paclitaxel + carboplatin and has completed 18 weeks of chemotherapy. In view of hypersensitivity to carboplatin, she is continued on palliative chemotherapy with paclitaxel. Her papulo-nodular lesions in her arm have disappeared, resulting in a scarred plaque (Figure 6).

## Discussion

Pathophysiology of cSCC includes a multistage process characterized by mutations in the genes involved in epidermal homeostasis, and by several alterations, such as epigenetic modifications, viral infections, or microenvironmental changes [4].

Other than histopathology, dermoscopy [5] and reflectance confocal microscopy [6] have enhanced the diagnostic accuracy of cSCC.

Surgical excision is the first-line treatment for invasive cSCC. Radiotherapy is the primary treatment for patients who are not ideal candidates for surgery. New systemic therapies, including chemotherapy, electrochemotherapy, and immunotherapy (such as cemiplimab), and epidermal growth factor receptor inhibitors, are used for metastatic and locally advanced cSCC [2].

The preventive measures used for cSCC include reduction in cumulative sun/UV radiation exposure, use of sunscreen, and prophylactic retinoids for high-risk patients [7,8].

Second primary cancer (SPC) is a term used to describe a new primary cancer that occurs in a person who has had cancer in the past. Various risk factors in SPCs associated with breast and skin cancers include age, gender, socio-cultural factors, radiotherapy, chemotherapy, and mutations in BRCAs, CDKN2A, VDK4, and BAP1.

The standardized incidence ratio (SIR) for primary cutaneous melanoma after breast carcinoma ranges from 1.16 to 5.13 [9]. Familial risk for secondary primary melanoma and SCC among breast cancer patients was found to be 1.32 and 1.35 in patients with a negative family history, and 1.97 and 3.47 in patients with a positive family history of breast cancer in a Swedish Family‐Cancer Database-based study, including 87,752 breast cancer patients [10].

Cutaneous metastasis (CM) mostly originates from breast cancer, melanoma, colorectal cancer, and lung cancer in adult women, and from lung cancer, melanoma, colorectal cancer, and prostate cancer in men [11]. Breast cancer metastasis to soft tissue has a better prognosis than metastasis to visceral organs or bone. Moreover, CMs from other internal malignancies carry a 4.3-fold increased relative risk of mortality compared to CMs from breast cancer [12]. CMs can be the presenting feature in a case of breast cancer [13].

## Conclusions

CM lesions are often misdiagnosed as benign lesions. Atypical or persistent nodular or non-healing ulcerative skin lesions, particularly in patients with a history of confirmed or suspected systemic malignancy, should undergo further examination (including radiological and histopathological investigations) to confirm the diagnosis and institute early treatment. As CM from breast cancer carries a good prognosis, a high index of suspicion, early diagnosis, and treatment are crucial in such cases.

## References

[1] Cives M, Mannavola F, Lospalluti L (2020). Non-melanoma skin cancers: biological and clinical features. Int J Mol Sci.

[2] Fania L, Didona D, Di Pietro FR (2021). Cutaneous squamous cell carcinoma: from pathophysiology to novel therapeutic approaches. Biomedicines.

[3] Kim JY, Kozlow JH, Mittal B, Moyer J, Olenecki T, Rodgers P (2018). Guidelines of care for the management of cutaneous squamous cell carcinoma. J Am Acad Dermatol.

[4] Nissinen L, Farshchian M, Riihilä P, Kähäri VM (2016). New perspectives on role of tumor microenvironment in progression of cutaneous squamous cell carcinoma. Cell Tissue Res.

[5] Zalaudek I, Argenziano G (2015). Dermoscopy of actinic keratosis, intraepidermal carcinoma and squamous cell carcinoma. Curr Probl Dermatol.

[6] Rishpon A, Kim N, Scope A (2009). Reflectance confocal microscopy criteria for squamous cell carcinomas and actinic keratoses. Arch Dermatol.

[7] Schmitt J, Seidler A, Diepgen TL, Bauer A (2011). Occupational ultraviolet light exposure increases the risk for the development of cutaneous squamous cell carcinoma: a systematic review and meta-analysis. Br J Dermatol.

[8] Green A, Williams G, Neale R (1999). Daily sunscreen application and betacarotene supplementation in prevention of basal-cell and squamous-cell carcinomas of the skin: a randomised controlled trial. Lancet.

[9] Jeyakumar A, Chua TC, Lam AK, Gopalan V (2020). The melanoma and breast cancer association: an overview of their ‘second primary cancers’ and the epidemiological, genetic and biological correlations. Crit Rev Oncol Hematol.

[10] Zheng G, Hemminki A, Försti A, Sundquist J, Sundquist K, Hemminki K (2019). Second primary cancer after female breast cancer: familial risks and cause of death. Cancer Med.

[11] Scolyer RA, Murali R, Thompson JF (2010). Cutaneous metastases. Foundations in Diagnostic Pathology, Dermatopathology.

[12] Mayer JE, Maurer MA, Nguyen HT (2018). Diffuse cutaneous breast cancer metastases resembling subcutaneous nodules with no surface changes. Cutis.

[13] Araújo E, Barbosa M, Costa R, Sousa B, Costa V (2020). A first sign not to be missed: cutaneous metastasis from breast cancer. Eur J Case Rep Intern Med.

